# HOTAIR Is a Potential Novel Biomarker in Patients with Congenital Heart Diseases

**DOI:** 10.1155/2018/2850657

**Published:** 2018-03-07

**Authors:** Yu Jiang, Hongdan Mo, Jing Luo, Suhong Zhao, Shuang Liang, Maomao Zhang, Jie Yuan

**Affiliations:** ^1^Department of Cardiology, The Second Affiliated Hospital of Harbin Medical University, Harbin 150001, China; ^2^The Key Laboratory of Myocardial Ischemia, Harbin Medical University, Ministry of Education, Heilongjiang Province 150001, China

## Abstract

**Objective:**

To investigate the expression of HOX transcript antisense RNA (HOTAIR) in cardiac tissues and plasma of patients with congenital heart diseases (CHDs).

**Methods:**

qRT-PCR was used to detect the expression of HOTAIR in right atrial appendage tissues of 16 patients with CHDs and 14 patients with rheumatic valvular heart diseases (RVHDs), as well as in plasma of 36 normal people and 90 patients with CHDs including 36 cases of ASD, 23 cases of VSD, and 31 cases of PDA. Besides, the proteins interacting with HOTAIR were obtained from databases.

**Results:**

The HOTAIR expression in cardiac tissues of CHDs group was significantly higher than that of the RVHDs group (*P* < 0.01). Compared with the control group, the expression of plasma HOTAIR in the ASD group, the VSD group, and the PDA group was all remarkably upregulated (*P* < 0.01), whereas there was no relationship between HOTAIR and pulmonary arterial hypertension and defects size. Databases show that HOTAIR is associated with polycomb repressive complex 2 (PRC2) which contributes to heart development.

**Conclusion:**

The levels of HOTAIR were increased in cardiac tissues and plasma of patients with CHDs. HOTAIR is a potential novel diagnostic biomarker in patients with CHDs.

## 1. Introduction

Congenital heart disease (CHD) is the most common type of congenital deformity and still represents an important noninfectious cause of death in children [1]. CHD is widely perceived to be a polygene heredity disease as a result of the complex interplay of genetic factors and environmental factors in the early development of embryos, but its pathogenic mechanism is still under research. A host of studies have shown that abnormal development of heart and vessels is associated with the mutation of many transcription factors including Nkx2.5, GATA4, and Tbx5 [2]. Long noncoding RNAs (lncRNAs) are defined as being noncoding RNA sequences of >200 nucleotides that can regulate target-gene expression, and they are closely related to malignant tumors, cardiovascular diseases, embryonic development, organism aging, and so on [3]. Mounting studies indicate that lncRNAs are new regulatory molecules involved in embryonic heart development after miRNAs [4, 5].

HOX transcript antisense RNA (HOTAIR) is the first transregulation lncRNA to be found. HOTAIR, which has a function of epigenetic regulation, has been shown to be relevant to many diseases, such as multiple cancers, osteoarthritis, and cardiovascular diseases [6]. Lots of studies have illustrated an important role of HOTAIR in cardiac diseases, including aortic valve calcification, cardiac hypertrophy, heart failure, and cardiac-associated sepsis [6–9]. However, little is known about the potential roles of HOTAIR in embryonic heart development. Therefore, in the present study we sought to determine the expression levels of HOTAIR in cardiac tissues and plasma of patients with CHDs, establishing some bases for further research about the function of HOTAIR on the embryonic heart development.

## 2. Materials and Methods

### 2.1. Study Population

The all-included cases with CHDs and rheumatic valvular heart diseases (RVHDs) were derived from patients who had been diagnosed at the Second Affiliated Hospital of Harbin Medical University, China, from November 2015 to June 2016. Human cardiac tissues were collected from 16 patients with CHDs and 14 patients with RVHDs who all required surgical treatment. Blood samples were collected from 90 patients with CHDs and 36 normal people. CHDs groups of blood samples were composed of 36 cases of atrial septal defect (ASD), 23 cases of ventricular septal defect (VSD), and 31 cases of patent ductus arteriosus (PDA). The inclusion criteria of CHDs are as follows: all cases were diagnosed with left-to-right shunt CHDs (ASD, VSD, and PDA), the diagnosis was verified by physical examination, X-ray, ECG, three-dimensional color Doppler echocardiography, and the corrective operation. The healthy individuals were from center of health examination, without a family history of CHDs and other congenital malformation. Patients with CHDs were diagnosed by two cardiology experts with three-dimensional color Doppler. Exclusion criteria were as follows: (i) complex CHDs; (ii) patients with acute infection, serious heart valve disease, stroke, malignant tumor, severe liver and kidney disease, and other severe systemic diseases; (iii) patients with use of immunosuppressant; (iv) patients with a history of recent surgery and muscle damage which change the levels of lncRNAs; (v) patients who provided incomplete information; (vi) patients who refused to participate in the study. The protocol was approved by the Ethics Committee of the Second Affiliated Hospital of Harbin Medical University (Number 246 Xuefu Road, Harbin, China) and informed consent was obtained from the parents or guardians. This study complies with the Declaration of Helsinki.

### 2.2. Definition of Pulmonary Arterial Hypertension (PAH)

PAH is evaluated according to the pulmonary artery systolic pressure (PASP) [10]: no pulmonary hypertension: PASP < 30 mmHg; pulmonary hypertension: PASP > 30 mmHg.

### 2.3. Dividing Standard of Defect Size (DS)

Defect size is the maximum value of each section. According to the ultrasonic measurement DS define [11, 12], for adult, small defect of VSD is smaller than 1 cm in diameter, small defect of ASD is smaller than 2 cm in diameter, medium to large defect of VSD is 1 cm or more in diameter, and medium to large defect of ASD is 2 cm or more in diameter. For child, small defect of VSD is smaller than 1 cm/m^2^ in diameter, small defect of ASD is smaller than 2 cm/m^2^ in diameter, medium to large defect of VSD is 1 cm/m^2^ or more in diameter, and medium to large defect of ASD is 2 cm/m^2^ or more in diameter (defect size and the ratio of surface area).

### 2.4. Tissue and Plasma Samples

Cardiac tissue specimens were acquired from right atrial appendage tissues of patients with CHDs and patients with RVHDs during surgical resection. Then the cardiac tissues were snap-frozen in liquid nitrogen and stored at −80°C immediately. Blood samples were collected from CHDs patients and healthy individuals and put into a test tube containing EDTA. Blood samples were centrifuged at 3000 rpm for 10 minutes immediately. The supernatant was transferred to new centrifuge tubes and recentrifuged at 12000 rpm for 10 minutes. All steps were performed at 4°C. Plasma was then collected, divided into aliquots, and stored frozen at −80°C before use.

### 2.5. RNA Isolation

Total plasma RNA was extracted from 500 *μ*l plasma using the Trizol (Invitrogen) according to the manufacturer's instruction and dissolved in 15 *μ*l of diethyl pyrocarbonate (DEPC) water. (i) Mix 500 *μ*l plasma and 800 *μ*l Trizol, and then let it sit for 5 minutes at room temperature. (ii) Then 160 *μ*l chloroform was added. (iii) The mixture was centrifuged at 12000 rpm for 15 minutes after 15 minutes' standing at room temperature. Then 800 *μ*l supernatant was collected. (iv) Mix the supernatant with 800 *μ*l isopropanol and let it sit for 4 hrs at −20°C. (v) The mixture was centrifuged at 12000 rpm for 15 minutes, then the supernatant was discarded, and 1 ml 75% anhydrous ethanol (DEPC water preparation) was added to the centrifuge tube. (vi) Repeat step (v). (vii) The mixture was centrifuged at 12000 rpm for 10 minutes, discarded the supernatant, dried the white RNA precipitation at the bottom of the tube at room temperature for 5 minutes, and dissolved it in 16 *μ*l DEPC water. The concentration and purity of RNA were detected by ultraviolet spectrophotometer. Total RNA of cardiac tissues was extracted from 50 mg tissues that were ground on ice and the following steps were also carried out according to the manufacturer's instruction.

### 2.6. Primer Design and Quantitative Real-Time PCR Analysis (qRT-PCR)

HOTAIR forward primer: 5′-GGTAGAAAAAGCAACCACGAAGC-3′, reverse primer: 5′-ACATAAACCTCTGTCTGTGAGTGCC-3′, product length is 170 bp. Reference GAPDH forward primer: 5′-CCGGGAAACTGTGGCGTGATGG-3′, reverse primer: 5′-AGGTGGAGGAGTGGGTGTCGCTGTT-3′, product length is 309 bp [13]. According to the manufacturer's protocol of the reverse transcription reagent kit (Bioneer company), 15 *μ*l total plasma RNA was used for cDNA synthesis. Next, we used AccuPower® 2×Greenstar™ qPCR Master Mix kit (Bioneer company) to detecte cDNA by CFX96TM real-time quantitative PCR detect system (Bio-Rad company). The reaction was performed in 20 *μ*l volume with 10 *μ*l 2×Greenstar Master Mix, 0.4 *μ*l Rox dye, 0.5 *μ*l each of forward and reverse primers, 7.2 *μ*l DEPC-treated water, and 2.0 *μ*l cDNA (2.0 *μ*l cDNA was replaced with 2.0 *μ*l DEPC-treated water in negative controls). Cycling parameters were as follows: 95°C for 10 minutes for initial denaturation followed by 40 cycles of 95°C for 15 sec and 61°C for 1 minute, then fluorescence signals were detected. The Ct value was defined as the cycle number at which the fluorescence (ΔRn) exceeded the threshold. A threshold of 0.20 was used as the default setting. Each sample's experiment was replicated 3 times; the average of the 3 results was the experimental result. The relative expression of target lncRNAs was determined by using the comparative cycle threshold (Ct) method (2^−ΔΔCT^); the melt curve analysis was used to confirm the specificity of amplification and the absence of primer dimers.

### 2.7. Statistical Analysis

The data was analyzed by SPSS 21.0 statistical software package. The normal distribution data was expressed by $\overset{-}{x}\pm s$. The nonnormal distribution data was expressed by median (M) and quartile (QR). And the data from categorical variables was analyzed by *χ*^2^ text. Distribution of continuous variables was tested by the single sample* K*-*S* test in nonparametric test. If the variables obey the normal distribution and homogeneity of variance, the two groups were compared by independent samples *t* test and multiple groups were compared by single factor analysis of variance. Otherwise, the mean was compared by nonparametric* K*-*W* test. The correlation test was performed by the Spearman correlation analysis. Images were rendered by GraphPad Prism 5.0 software. Bilateral *P* < 0.05 was considered statistically significant.

### 2.8. Bioinformatic Analysis

Proteins interacting with HOTAIR were obtained from LncRNA and Disease Database (LncRNADisease) (http://www.cuilab.cn/lncrnadisease) up until July 2016 with “HOTAIR” as the search term and the interaction between proteins was downloaded from Human Protein Reference Database (HPRD) (http://www.hprd.org/) up until July 2016.

## 3. Results

### 3.1. General Conditions

16 patients with CHDs were enrolled in tissue research including 9 males and 7 females, and 14 patients with RVHDs were composed of 6 males and 8 females. There was no statistical difference in composition of gender (*χ*^2^ = 0.536, *P* = 0.464). However, there was a significant difference in composition of age as it was difficult to match the age between patients with CHDs and patients with RVHDs (*F* = 0.068, *P* < 0.05) (Table 1). 90 patients with CHDs were enrolled in plasma research including 36 cases of ASD consisted of 16 males and 20 females, 23 cases of VSD composed of 10 males and 13 females, and 31 cases of PDA made up of 9 males and 22 females. 36 cases of healthy group were consisted of 17 males and 19 females. There were no statistical differences between composition of gender and age (*χ*^2^ = 2.638, *P* = 0.451 and; *H* = 6.050, *P* = 0.109) (Table 2).

### 3.2. Results of Real-Time Quantitative PCR

**Figure 1:**
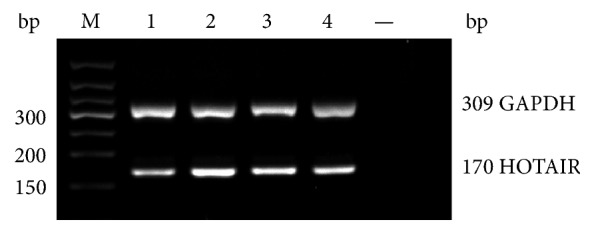
*Electrophoresis results of real-time quantitative PCR HOTAIR and GAPDH products*. 1: normal group. 2: ASD group. 3: VSD group. 4: PDA group. —: negative controls. M: DNA ladder.

**Figure 2:**
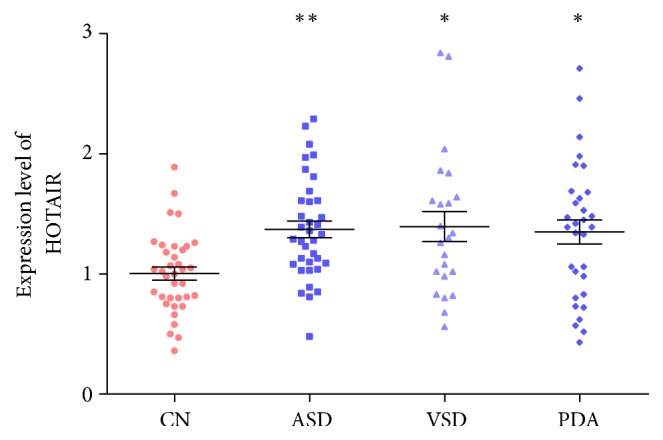
*HOTAIR were detectable in plasma from CHDs patients and healthy controls*. ^*∗*^Compared with control group, *P* < 0.05; ^**∗∗**^compared with control group, *P* < 0.01.

The light density was detected after total RNA was isolated from cardiac tissues and plasma. The purity was good; meanwhile, the *D* (260 nm)/*D* (280 nm) was between 1.8~2.0. At the same time, there was no amplification in negative control. 2% agarose gel electrophoresis was used to detect the PCR products, which showed that specific purpose strips of HOTAIR and GAPDH separately appeared in 170 bp and 309 bp (Figure 1). The average levels of HOTAIR expression in cardiac tissues of patients with CHDs and patients with RVHDs as well as in plasma of the ASD group, the VSD group, the PDA group, and the normal group were (0.111 ± 0.015), (0.055 ± 0.007), (1.37 ± 0.42), (1.39 ± 0.60), (1.35 ± 0.57), and (1.00 ± 0.33), respectively. The HOTAIR expression in cardiac tissues of patients with CHDs was significantly higher than that of patients with RVHDs (*P* = 0.002). Compared with the normal group, the expression of plasma HOTAIR in CHDs groups was marked increased (*P* = 0.001). Among the CHDs groups, the expression levels of plasma HOTAIR in the ASD group (*P* = 0.002), the VSD group (*P* = 0.040), and the PDA group (*P* = 0.020) were all remarkable upregulated. Among these groups, the ASD group increased predominantly (Figure 2). In addition, we also analyzed the relevance between age and the expression of plasma HOTAIR in the ASD group. The age range was from 1 to 53 and the average age was 24.03 ± 16.53. Result showed that there was no statistically significant difference between age and the expression of HOTAIR (*P* > 0.05).

### 3.3. Comparison of HOTAIR Levels in ASD and VSD Patients with Different DS

**Figure 3:**
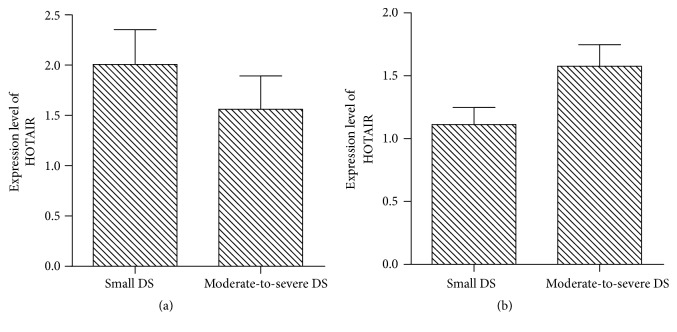
Comparison of the expression of HOTAIR in ASD (a) and VSD (b) patients with different DS.

According to the defect size of ASD and VSD measured via ultrasound, patients were divided into two groups. In ASD group, HOTAIR expression in plasma was not statistically significant (*t* = 1.876, *P* = 0.069) between 14 patients with small defect (1.53 ± 0.43) and 22 patients with moderate to severe (1.27 ± 0.39). In VSD group, HOTAIR expression in plasma was not statistically significant (*t* = 1.907, *P* = 0.070) between 9 patients with small defect (1.11 ± 0.41) and 14 patients with moderate to severe (1.57 ± 0.65) (Figure 3).

### 3.4. Comparison of the Expression of HOTAIR in CHDs Patients with or without PAH

**Figure 4:**
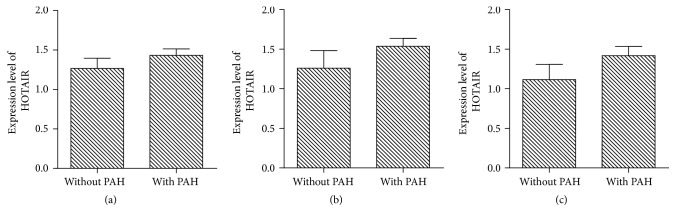
Comparison of the expression of HOTAIR in ASD (a), VSD (b), and PDA (c) patients with or without PAH.

In ASD group, HOTAIR levels were (1.27 ± 0.47) in 13 cases of without PAH and (1.43 ± 0.39) in 23 cases of with PAH. The difference revealed no statistical significance (*t* = 1.128, *P* = 0.267). In VSD group, HOTAIR levels were (1.26 ± 0.77) in 12 cases of without PAH and (1.54 ± 0.32) in 11 cases of with PAH. The difference revealed no statistical significance (*t* = 1.113, *P* = 0.278). In PDA group, HOTAIR levels were (1.12 ± 0.51) in 7 cases of small defect without PAH (1.42 ± 0.57) in 24 cases of small defect with PAH. The difference revealed no statistical significance (*t* = 1.254, *P* = 0.220) (Figure 4).

### 3.5. Proteins Interacting with HOTAIR and the Interaction between Proteins

**Figure 5:**
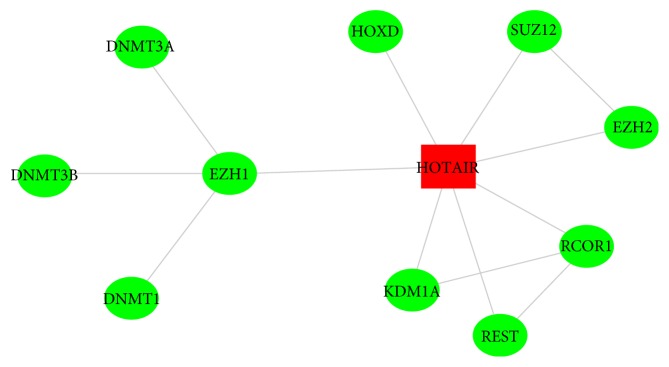
The proteins interacting with HOTAIR and the interaction between proteins.

The interacting proteins contain EZH1, EZH2, SUZ12, REST, KDM1A (LSD1), RCOR1 (COREST), and HOXD. Then a network of HOTAIR and its interacting proteins as well as a network of the interaction between proteins can be constructed (Figure 5).

## 4. Discussion

The human HOTAIR locates in the HOXC cluster on chromosome 12. The HOTAIR gene is transcribed in an antisense direction relative to its flanking HOXC11 and HOXC12 genes. Its principal transcript is a 2364 bp RNA transcribed from a 6449 bp gene locus and composed of 6 exons. HOTAIR was coexpressed with HOXC gene cluster [14]. Human HOX gene cluster contains four subfamilies: HOXA, HOXB, HOXC, and HOXD that the proteins they encode have a conserved homeodomain, which is related to human embryonic development and plays a pivotal role in regulating tissue differentiation and organ sculpting [15]. Studies have shown that HOTAIR was involved in cancer invasion and metastasis [16, 17]. A previous study has proved that HOTAIR was important to modulate proliferation and differentiation of mesenchymal stem cells [18]. Thus, HOTAIR plays an indispensable part in various physiological and pathological processes by epigenetic regulation.

Because the age range of patients with CHDs is widely distributed, including all ages from newborn to the survived crowd, and it is difficult to obtain myocardial tissue samples from normal population, we chose to detect the expression of HOTAIR in right atrial appendage tissues of patients with CHDs and patients with RVHDs. The results of the present study indicated that the HOTAIR expression in cardiac tissues of patients with CHDs was significantly higher than that of patients with RVHDs. In order to match the composition of age and expand the number of samples, we also chose to determine the expression of plasma HOTAIR between patients with CHDs and healthy individuals. In our study, plasma HOTAIR expression was also detected to be upregulated in CHDs. Notably, ASD group was most significantly increased. Besides, there was no correlation between age and HOTAIR expression. There was no significant relationship between plasma HOTAIR and defects size and PAH to be found in CHDs patients.

PRC2 is mainly composed of three core subunits EED, SUZ12, and EZH2 [19]. The biological analysis shows that proteins interacting with HOTAIR include EZH2, SUZ12, and KDM1A (LSD1), so HOTAIR has the function of acting as a scaffold for histone methylase (PRC2) and histone demethylase (LSD1). Many malignant tumor experiments have demonstrated that HOTAIR altered histone H3 lysine 27 methylation by recruiting PRC2 to suppress target genes and control Wnt/*β*-catenin [13, 20]. Previous studies have shown that Wnt signaling pathway played a vital role in regulating cell proliferation, migration, and heart development. Abnormal activation of Wnt signaling pathway caused by genetic and epigenetic changes was associated with the pathogenetic mechanism of CHDs [21]. Thus, as a key Wnt signaling pathway regulating factor, HOTAIR may play an important role in cardiac development. Moreover, PRC2 has methyltransferase activity that can regulate the expression of noncardiac genes such as INK4a/b, ISL1, and Six1 and cardiac transcription factors such as GATA4 by catalyzing histone H3K27 and DNA methylation, thereby having an impact on embryonic heart development [19, 22]. On the other hand, HOTAIR might adjust the level of the intracellular calcium by repressing the expression of Ca_V1.2_ and interfere with the occurrence and development of many diseases [23]. Recent studies have found that calcium channel blockade agent (nifedipine) inhibits the early embryonic mesoderm multipotent stem cells which differentiate into cardiomyocytes, and the expression of transcription factors GATA4 and Nkx2.5 was significantly decreased [24]. The effect of HOTAIR on cardiac development may be related to this similar function. For these reasons, we speculate that HOTAIR may be closely related to cardiac development and CHDs. HOTAIR overexpression may affect embryonic heart development by recruiting PRC2 to epigenetic regulate gene expression and influencing the signaling pathways, for instance, Wnt signaling pathway and calcium channel. The exact mechanism of HOTAIR on embryonic heart development is expected to go on in the near future.

To our knowledge, we have demonstrated for the first time that HOTAIR was highly expressed in cardiac tissues and plasma of patients with CHDs and it shows that HOTAIR may epigenetically regulate embryonic heart development by recruiting PRC2. These findings indicate that HOTAIR may be a potential novel biomarker in patients with CHDs. We hope this study can provide a new clue for further investigation into the mechanism of embryonic heart development.

## Figures and Tables

**Table 1:** General conditions of patients with CHDs group and patients with RVHDs group.

Characteristics	CHDs (*n* = 16)	RVHDs (*n* = 14)	Statistic	*P*
Gender (male/female)	9/7	6/8	*χ* ^2^ = 0.536	0.464
Age ($\overset{-}{x}$ ± *s*, years)	11.94 ± 1.42	45.50 ± 1.58	*F* = 0.068	<0.05
Expression of HOTAIR	0.111 ± 0.015^*∗*^	0.055 ± 0.007	*F* = 3.171	0.002

^*∗*^Compared with RVHDs group, *P* < 0.05; CHDs: congenital heart diseases; RVHDs: rheumatic valvular heart diseases.

**Table 2:** General conditions of patients with CHDs group and control group (CN).

Characteristics	CN (*n* = 36)	ASD (*n* = 36)	VSD (*n* = 23)	PDA (*n* = 31)	Statistic	*P*
Gender (male/female)	17/19	16/20	10/13	9/22	*χ* ^2^ = 2.638	0.451
Age ($\overset{-}{x}\pm s$, years)	23.08 ± 14.75	24.03 ± 16.52	15.30 ± 16.31	21.48 ± 19.77	*H* = 6.050	0.109
DS ($\overset{-}{x}\pm s$, cm)	—	2.27 ± 0.90	1.17 ± 0.76	0.69 ± 0.44	—	—
PASP ($\overset{-}{x}\pm s$, mmHg)	—	34.21 ± 9.11	38.75 ± 17.03	50.90 ± 27.25	*H* = 4.341	0.114
LEVF ($\overset{-}{x}\pm s$, %)	—	62.56 ± 1.93	62.87 ± 1.14	61.70 ± 4.87	*F* = 1.047	0.355
Expression of HOTAIR	1.00 ± 0.33	1.37 ± 0.42^*∗∗*^	1.39 ± 0.60^*∗*^	1.35 ± 0.57^*∗*^	*H* = 15.665	0.001

^*∗*^Compared with CN group, *P* < 0.05; ^*∗∗*^compared with CN group *P* < 0.01; LEVF: left ventricular ejection fraction; ASD: atrial septal defect; VSD: ventricular septal defect; PDA: patent ductus arteriosus.
